# Electrochemically Reduced Graphene Oxide-Based Screen-Printed Electrodes for Total Tetracycline Determination by Adsorptive Transfer Stripping Differential Pulse Voltammetry

**DOI:** 10.3390/s20010076

**Published:** 2019-12-21

**Authors:** Anabela S. Lorenzetti, Tania Sierra, Claudia E. Domini, Adriana G. Lista, Agustin G. Crevillen, Alberto Escarpa

**Affiliations:** 1 Department of Analytical Chemistry, Physical Chemistry and Chemical Engineering, University of Alcala, E-28871 Alcala de Henares, Spain; anabela.lorenzetti@gmail.com (A.S.L.); tania.sierra@edu.uah.es (T.S.); 2 INQUISUR, Department of Chemistry, Universidad Nacional del Sur (UNS)-CONICET, Av. Alem 1253, Bahía Blanca 8000, Argentina; tino_alcarria@hotmail.com; 3 Department of Analytical Sciences, Faculty of Sciences, Universidad Nacional de Educación a Distancia (UNED), E-28040 Madrid, Spain; agustingcrevillen@ccia.uned.es; 4 Chemical Research Institute “Andrés M. del Río” (IQAR), University of Alcalá, E-28805 Alcalá de Henares, Spain

**Keywords:** tetracycline, adsorption, graphene, adsorptive and electrochemical selectivity, disposable sensors, sample screening

## Abstract

Disposable electrochemically reduced graphene oxide-based (ERGO) screen-printed electrodes (SPE) were developed for the determination of total tetracyclines as a sample screening approach. To this end, a selective adsorption-detection approach relied on adsorptive transfer stripping differential pulse voltammetry (AdTDPV) was devised, where the high adsorption capacity and the electrochemical properties of ERGO were simultaneously exploited. The approach was very simple, fast (6 min.), highly selective by combining the adsorptive and the electrochemical features of tetracyclines, and it used just 10 μL of the sample. The electrochemical sensor applicability was demonstrated in the analysis of environmental and food samples. The not-fully explored AdTDPV analytical possibilities on disposable nanostructured transducers become a new tool in food and environmental fields; drawing new horizons for “in-situ” analysis.

## 1. Introduction

Graphene is a carbon allotrope composed of a single layer of carbon with partially filled sp^2^-orbitals above and below the plane of the sheet. This nanomaterial has attracted the attention of scientific community due to its amazing features, such as large surface area (2630 m^2^/g for a single layer), high mechanical strength, and high elasticity (Young’s modulus ≈ 1100 GPa); and excellent thermal (5 × 10^3^ Wm^−1^ K^−1^) and electrical conductivity (charge carrier mobility > 200,000 cm^2^ V^−1^ s^−1^ for freely suspended graphene) [1].

Graphene has been successfully used in all analytical steps: sample preparation, separation, and detection [1,2,3]. Regarding sample preparation, graphene has been applied in solid phase extraction and solid phase microextraction because of its excellent adsorption ability [4,5]. These applications were mainly focused on the extraction of benzene derivatives, which can easily interact, by π–π stacking with graphene thanks to its large electron delocalization. For example, benzene derivatives such as polycyclic aromatic hydrocarbons [6], tetracyclines [7], sulphonamides antibiotics [8], among others, were extracted by graphene-based sorbents. Respect to the use of graphene for direct electrochemical sensing, this nanomaterial provides (i) high heterogeneous electron transfer rate, (ii) electrocatalytic properties against several molecules (due to the high number of atoms localized in the edges/defects), and (iii) high surface to volume ratio that increases sensitivity [2,9]. Taking advantage of this features, a plenty of electrochemical sensors were developed exclusively based on graphene for direct detection of H_2_O_2_, ascorbic acid, uric acid, dopamine, and several phenol-containing compounds, among others [9,10]. Furthermore, the combination of screen-printed electrode (SPE) technology and graphene is a cutting-edge strategy for “in situ” analysis and for developing new point-of-care testing (POC) devices. SPE technology provides reliable single-use devices, which can be easily modified [11], and graphene provides an improvement of electroanalytical performance [10].

**Figure 1:**
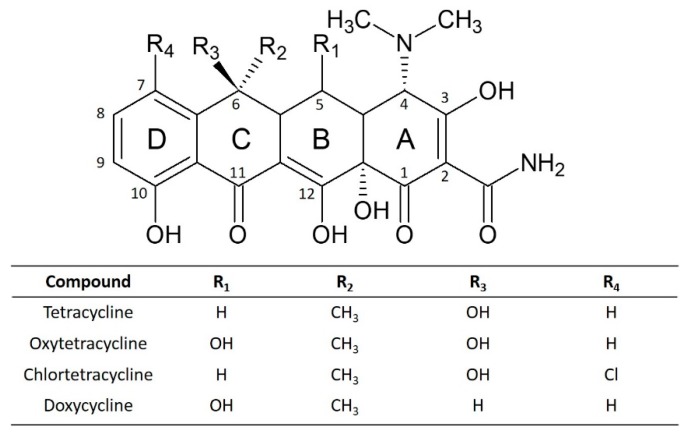
Chemical structures of studied tetracyclines.

On the other hand, tetracyclines are a kind of antibiotics with a chemical structure derived from a hydronaphthacene containing four fused rings (see Figure 1). All of them also contain a phenol group that is electroactive [12]. This group of antibiotics has been exhaustively employed in human and animal medicine and also as an additive in animal feed because of its broad spectrum antimicrobial activity and low cost [13]. Within this family, tetracycline (TET), oxytetracycline (OTC), chlortetracycline (CTC), and doxycycline (DOX) are the most prescribed in the veterinary world [14]. The intensive use and abuse of antibiotics, in general, and tetracyclines, in particular, has led to an increase of bacterial strains resistant to antimicrobial agents, causing a public health alarm worldwide [15]. For these reasons, a lot of countries legislated to establish maximum residual limits (MRL) in animal-derived food [14]. Moreover, the presence of tetracyclines is not limited to foodstuff but also to environmental through wastewater because sewage treatment plants cannot fully remove them [15].

Several analytical techniques have been successfully employed for determination of residual tetracyclines such as separation techniques (HPLC, CE), spectrophotometry or ELISA [14]; however, they present some drawbacks such as a complicated sample pretreatment process and/or the need of a trained lab technician [16]. In this sense, sensors are an attractive alternative or complementary analytical tools for tetracycline detection because of their inherent advantages such as high selectivity, rapid detection, and in-situ applications [17]. In fact, sensors are ideal candidates for developing sample-screening methods, namely methods used to detect the presence of an analyte or class of analytes at the level of interest. A screening method is the first method that is applied to sample analysis with the purpose to confirm the presence or absence of antibiotic residues. This procedure should be simple, fast, cheap, and sensitive. In addition, if an extraction is needed, it should be very simple and quick [18].

Graphene based electrochemical sensors have been used for TET determination obtaining good sensitivities [19,20]. Kesavan et al. detected TET in urine in presence of uric acid (UA), which is highly electroactive, using polymelamine/ERGO modified electrode [19]. Sun et al. determined TET in water samples using a Graphene/L-Cysteine composite film [20]. Both papers used the DPV technique and carried out an interference study mainly focused on small inorganic and organic ions but not on electroactive compounds. There is another interesting work, in which a carbon paste electrode was fabricated combining multi-walled carbon nanotube and graphene oxide for TET detection [21]. An adsorptive stripping differential pulse voltammetry (AdSDPV) was employed; first, TET was electro-accumulated and then, it was analyzed. The sensor was applied to different samples (river water, artificial urine without uric acid, and pharmaceutical samples).

However, there is a modification of AdSDPV technique called adsorptive *transfer* stripping differential pulse voltammetry (AdTDPV) which has not been used for TET determination yet. This consists of three steps: (i) electrode is dipped in the sample and the analyte is adsorbed, (ii) the electrode is taken out from the sample and rinsed, and (iii) the electrode is dipped in the background electrolyte (analyte is transferred to another solution). This technique provides two advantages: (i) low adsorbed compounds can be easily washed away during the transfer, so their interference is suppressed; (ii) the adsorption medium and the background electrolyte can be different allowing analyst to optimize background electrolyte conditions [22].

In this work, graphene oxide was electrochemically reduced on SPEs (ERGO-SPE). These electrodes were applied for the detection of tetracyclines in food and environmental samples, by using a selective adsorption-detection approach based on adsorptive transfer stripping differential pulse voltammetry (AdTDPV). This method creatively combines the selective adsorption ability of graphene along with its high electrochemical performance, on board on disposable screen-printed carbon electrodes (SPCE); simplifying in extreme the electrochemical sensor construction.

## 2. Material and Methods

### 2.1. Reagents and Sample

Tetracycline hydrochloride (TET), chlortetracycline hydrochloride (CTC) doxycycline hydrochloride (DOX), oxytetracycline hydrochloride (OTC), hexaammineruthenium(II) chloride, hexaammineruthenium(III) chloride, graphene oxide (GO, ref:763705) and trichloroacetic acid were purchased from Sigma-Aldrich (https://www.sigmaaldrich.com). Disodium hydrogen phosphate and sodium dihydrogen phosphate were purchased from Panreac (http://www.panreac.com). Ascorbic acid was purchased from Fluka Chemie (via Sigma-Aldrich, Darmstadt, Germany). All solutions were prepared in Milli-Q water (https://www.merckmillipore.com). Skim milk was acquired in a Spanish market. River water samples were collected from Henares River (Spain).

### 2.2. Instrumentation

Potentiostat Autolab PGSTAT204 (https://www.metrohm-autolab.com) was used for the electrochemical analysis and for ERGO-SPE fabrication. This instrument was controlled by Nova 1.10 software. Screen-printed electrodes (SPEs) (DRP-110, http://www.dropsens.com) composed of carbon working electrode (4 mm), carbon counter electrode and silver reference electrode, were employed. This kind of SPEs works as an electrochemical cell, which needs a minimum volume of 50 μL. Surface morphologies of modified and unmodified electrode were studied by scanning electron microscopy (SEM) (DSM 950, Zeiss, Germany, www.zeiss.com).

### 2.3. Procedures

#### 2.3.1. Electrochemical Sensor Fabrication

Electrochemically reduced graphene oxide-based screen printed electrodes (ERGO-SPE) were fabricated upon a disposable SPE. Firstly, GO was dispersed in phosphate buffer 50 mM pH 6.0 to obtain a final concentration of 0.7 mg mL^−1^. Dispersions were sonicated using an ultrasonic bath. 50 µL of this solution were dropped on the surface of the working electrode and then, cyclic voltammetry technique (from 0.1 to −1.5 V with a scan rate of 100 mV s^−1^ for 20 cycles) was applied to obtain the ERGO-SPEs [23].

#### 2.3.2. Electrochemical Measurements

The amount of oxygen containing species on different electrodes surface (SPE, ERGO-SPE 10 cycles and 20 cycles) were estimated by monitoring the oxygen species reduction using cyclic voltammetry (conditions: one cycle at 100 mV s^−1^ from 0 V to −1.5 V) using 50 mM phosphate buffer pH 6.0, previously, deoxygenated by bubbling with N_2_ for 20 min [24].

Determination of tetracyclines (TETs) was carried out at room temperature, employing adsorptive transfer differential pulse voltammetry (AdTDPV) (optimized parameters: pulse amplitude of 125 mV, pulse time 0.05 s, interval time 0.3 s, step potential 8 mV). All measurements were performed using 50 mM phosphate buffer pH 6.0. The process began by dropping 10 μL of working solution or sample onto the working electrode and, then, it is kept at open circuit potential for 5 min to allow analytes to adsorb on the electrode surface. After that, the electrode was rinsed with water for 1 min followed by the addition of 50 μL of the background electrolyte on the electrode in order to carry out the DPV measurement.

#### 2.3.3. Measurements of Electrode Active Surface by Using a Randles–Sevcik Equation

The Randles–Sevcik equation (at 25 °C) is followed by a reversible process in cyclic voltammetry:

Ip =2.69 ×105n32 A C Do12v12,

Ip being the anodic peak current, n the electron transfer number, A the electrode surface area, *C* the concentration, *D* the diffusion coefficient of redox probe, and *v* the scan rate. It is possible to measure the electrode surface area calculating the slope of Ip vs. v12 plot with the use of a reversible redox probe. In this way, cyclic voltammograms of 1 mM [Ru(NH_3_)_6_]Cl_3_ in 0.5 M KNO_3_ solution were recorded at different scan rates (from *v* = 0.01 to *v* = 0.1 V s^−1^) from −0.8 to +0.6 V using SPE and ERGO-SPEs (*n* = 3) (number of transferred electrons = 1 and *Do* = 7.74 × 10^−6^ cm^2^ s^−1^).

### 2.4. Sample Preparation

The applicability of the proposed electrode was assessed in river water and skim milk. River water was analyzed as collected (no sample treatment). Milk samples were treated as follows [25]: 0.4 mL skim milk was placed into a 1.5 mL microtube, and diluted to 1.0 mL with water. Then, 0.2 mL of 10% trichloroacetic acid was added and mixed by vortex during 1 min to favor protein precipitation. Next, the mixture was centrifuged at 13,000 rpm for 10 min. Afterwards, 0.5 mL of the supernatant was transferred into an Amicon^®^ centrifugal filter unit (Ultra-0.5 mL, Ultracel-10K, ref UFC501024) and centrifuged at 12,800 rpm for 20 min (MiniSpin^®^, Eppendorf, Hamburg, Germany) to eliminate completely non-precipitated proteins. The final solution was used for detection (see Section 2.3.2).

## 3. Results and Discussion

For screening purposes, it is not important to identify what tetracycline specifically occurs in the sample but to detect the presence of any member of this kind of antibiotics at the level of regulatory maximum residual limits. Therefore, the development of a method for total tetracycline determination is a pertinent approach.

In this sense, AdTDPV allows analysts to improve the selectivity of electrochemical sensors by exploiting the adsorption properties of the sensing surface. To demonstrate the usefulness of this technique as sample screening tool, it is necessary to perform: (i) a characterization of the sensor surface, (ii) a comparison of analyte response between the corresponding adsorptive technique (AdTDPV) with the non-adsorptive (DPV) one, and (iii) a study of the analytical performance of sensor (with special attention to the potential interferences).

### 3.1. ERGO-SPE Characterization

Two different ERGO-SPEs were prepared varying the number of cycles applied during GO reduction to study their analytical performance for TET determination. Jampasa et al. used 16 cycles for ERGO-SPE fabrication [23]; consequently, we used 10 and 20 cycles (termed as ERGO-10 and ERGO-20, respectively). Since AdTDPV was selected for total tetracycline determination, the influence of the number of cycles was studied accordingly to (i) the electrode active surface and (ii) the amount of oxygen species on electrode surface, which may generate different electrochemical and adsorptive responses toward tetracyclines.

Due to AdTDPV measures, only analytes adsorbed on the electrode, the larger the surface of the electrode, the greater the signal. For this reason, the active surface of ERGO-SPEs and SPE (control) were measured by means of cyclic voltammetry using the Randles–Sevcik equation. The resulting surface areas of SPE, ERGO-10, and ERGO-20 were 0.215 cm^2^, 0.333 cm^2^, and 0.753 cm^2^, respectively. ERGO-20 exhibited higher active surface in comparison with SPE and ERGO-10.

**Figure 2:**
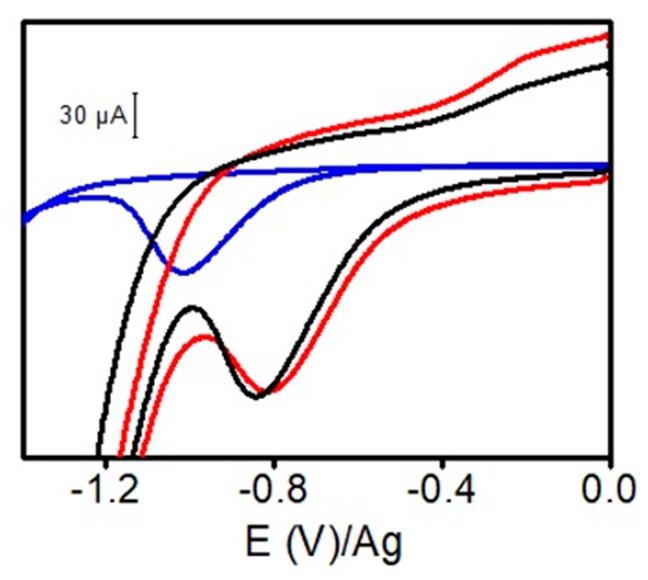
Cyclic voltammograms of phosphate buffer 50 mM pH 6.0 using different electrodes: SPE (blue line), ERGO-10 (red line), and ERGO-20 (black line). Conditions: scan rate 100 mV s^−1.^

Next, the amount of oxygen containing species on electrode surface was estimated by cyclic voltammetry (see Figure 2). Both ERGO-10, 20 yielded a reduction peak around −0.80 V with a similar area so we inferred that the number of cycles used for ERGO preparation does not affect the amount of oxygen species on the electrode surface. However, SPE yielded a reduction peak (−1.0 V) smaller than those obtained in ERGO-SPEs. Thus, ERGO-SPEs presented a higher amount of oxygen species on the surface than SPE. On the other hand, the different reduction potential between SPE (−1.0 V) and ERGO-SPEs (−0.80 V) may be related with the presence of different kinds of oxygen containing species.

**Figure 3:**
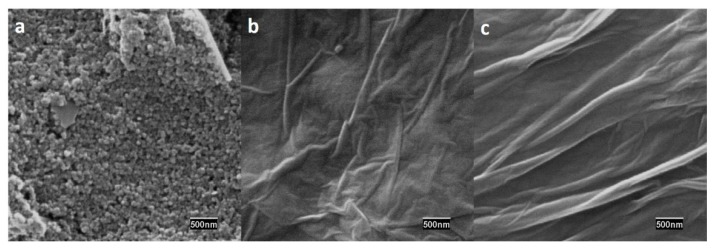
SEM images of (**a**) SPE, (**b**) ERGO-10, (**c**) ERGO-20 (scale bar 500 nm).

The surface morphology of both ERGO-SPEs and SPE was also studied by SEM. As it can clearly be seen in Figure 3b,c, an ERGO film was formed on the SPE substrate. The typical wrinkling surface of ERGO was obtained due to the electrochemical reduction process of GO and the consequent deoxygenation [26].

### 3.2. Electrochemical Behavior of Tetracyclines at ERGO-SPE by AdTDPV

**Figure 4:**
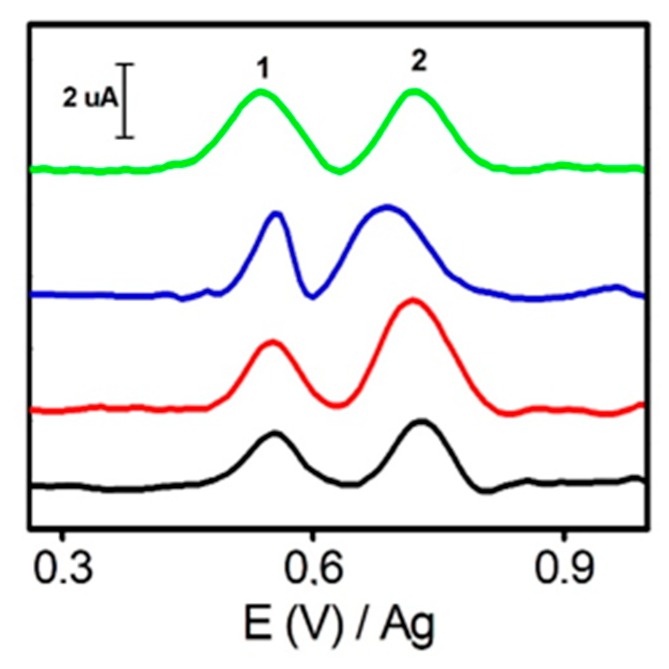
AdTDPV voltammograms corresponding to 40 μM of TET (black), CTC (red) and DOX (blue) and OTC (green) using ERGO-20. Conditions: phosphate buffer 50 mM pH 6, pulse amplitude 125 mV, pulse time 0.05 s, interval time 0.3 s, step potential 8 mV, adsorption time 5 min.

An electrochemical response of individual tetracyclines (TET, OTC, CTC and DOX) was found to be very similar by AdTDPV, showing two well-defined oxidations peaks (around E = +0.55 V and +0.75 V, see Figure 4). Consequently, tetracycline (TET) was chosen as a model for the next experiments. These analytes were previously studied by cyclic voltammetry; however, the obtained sensitivity and peak resolution were worse than those obtained by AdTDPV (see Figure S1, electronic supplementary material ESM). Then, TET was analyzed by AdTDPV using SPE and ERGOs-10, 20. To demonstrate conceptually the use of AdTDPV, differential pulse voltammetry (DPV) was also explored with comparative purposes. Table 1 summarizes the most important data from analysis of peak 1 of TET at the most selective potential around +0.55 V. The peak intensity obtained by DPV is much higher using SPE than using ERGO. However, this situation reversed when AdTDPV is used. ERGO electrodes yield higher signals than SPE because of the higher adsorption capacity of the formers and the low affinity of TET on a carbon SPE surface. These results disclose the strong π–π stacking between the π–conjugate rings of TET and graphene. Finally, ERGO-20 showed the highest peak intensity by AdTDPV so it was chosen for the following experiments. The suitability of this selection will be confirmed in the next sections accordingly to selectivity studies.

### 3.3. Analytical Performance of AdTDPV-ERGO-SPE Sensor for Total Tetracycline Determination and Sample Analysis

Then, pulse amplitude, adsorption time, and pH were optimized to obtain the highest sensitivity at the most selective oxidation potential of approx. +0.55 V (peak 1). Firstly, the influence of pulse amplitude on the AdTDPV peak signal was evaluated (see Figure S2, ESM). By applying voltage amplitudes from 25 mV to 150 mV, the maximum peak intensity was obtained at 125 mV, so it was selected as the optimal value.

The influence of adsorption time of tetracyclines on the ERGO-SPE surface was also tested using 0.5 mM TET (1, 2, 5 and 10 min). The peak intensities obtained for 1, 2, 5, and 10 min. were 1.34 µA, 1.69 µA, 2.97, and 3.68 µA, respectively. A small increase in the signal was obtained using 10 min respect to 5 min so that the latter was set as the optimal value as a compromise between the good sensitivity and shorter analysis times.

Finally, a pH study was performed from 3.5 to 9.0 using 0.5 mM TET in a 50 mM phosphate buffer (see Figure S3). It is well known that the oxidation mechanism of TET is pH dependent (the lower pH the higher oxidation peak) [12]. However, only adsorbed TET molecules give a signal in AdTDPV. The maximum signal occurred at pH 6.0, very close to the isoelectric point of TET (pI 5.4) [27]. This means that hydrophobic interactions are dominant in the adsorption process of TET on the ERGO surface.

Linear calibration plot for each tetracycline were built (*n* = 3), obtaining linear ranges from 20 µM to 80 µM (*n* = 4, see Figure S4). This short linear ranges are typical of organic compounds analyzed by adsorptive stripping voltammetry techniques [28]. To develop a method for total tetracycline determination, it is mandatory to demonstrate that the method sensitivity (calibration slopes) are similar for all tetracyclines. Therefore, an analysis of variance (ANOVA) test was carried out to compare the slopes of the different linear regression plots. The slopes showed non-statically significant differences (*p* < 0.05), so the calibration equation from any of tetracyclines can be used to estimate total tetracyclines.

**Figure 5:**
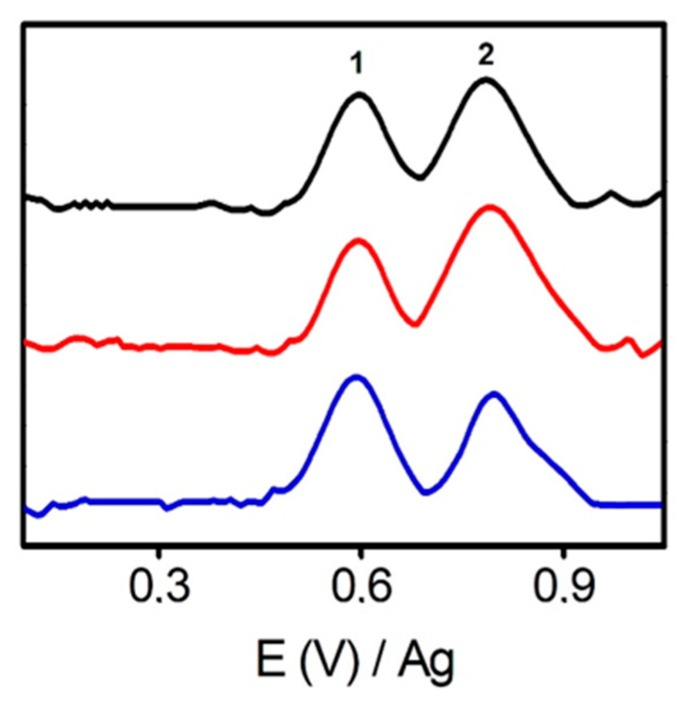
AdTDPV voltammograms corresponding to different mixtures of tetracyclines using ERGO-20. Mixture 1 (Black line): TET = 20 μM, OTC, CTC, DOX = 15 μM; Mixture 2 (Red line): TET = 10 μM, OTC = 5 μM, CTC = 25 μM, DOX = 25 μM; Mixture 3 (Blue line): TET = 30 μM OTC = 25 μM, CTC = 5 μM, DOX = 5 μM. Conditions: phosphate buffer 50 mM pH 6, pulse amplitude 125 mV, pulse time 0.05 s, interval time 0.3 s, step potential 8 mV and, just for AdTDPV, adsorption time 5 min.

To reinforce our approach, mixtures with different concentrations of tetracyclines were analyzed by triplicate using our method (see Figure 5). Quantitatively, the three mixtures assayed (total tetracyclines concentration 65 μM: mixture 1: TET = 20 μM, OTC, CTC, DOX = 15 μM; mixture 2: TET= 10 μM, OTC = 5 μM, CTC = 25 μM, DOX = 25 μM; mixture 3: TET = 30 μM OTC = 25 μM, CTC = 5 μM, DOX = 5 μM) gave peak intensities of 1.25 ± 0.11 μA, 1.14 ± 0.14 μA and 1.37 ± 0.19 μA, respectively. These results did not show statistically significant differences (*p* < 0.05).

In this sense, TET was selected as standard for total tetracycline determination and a new calibration plot was built by averaging the previous three independent calibration curves for TET (Y = (2.11 ± 0.25) × 10^−8^ X − (2.09 ± 1.39) × 10^−7^, where X was expressed as µM and Y as A, r = 0.990). The limit of detection (LOD) was 12 µM (calculated using standard deviation of intercept, 3 S/N criterion). Inter-electrode precision was also evaluated by measuring an 80 µM TET solution using five different ERGO-SPE, obtaining an RSD of 18%. The main reason of this variability is the differences in active surface between ERGO-SPEs.

Although in other related works LODs were lower [19,20,21], they used glassy carbon electrodes (GCE) or carbon paste electrodes (CPE) which are not disposable so that the electrodes must be cleaned and modified between analysis. Our approach takes the advantages of the use of single-use disposable approach with just 10 µL of sample.

To demonstrate conceptually the suitability of the AdTDPV-based sensor for TET determination, ascorbic acid, phenol, and uric acid were explored as target analytes of high miscellanea significance with a wide distribution. DPV was also used as control.

**Figure 6:**
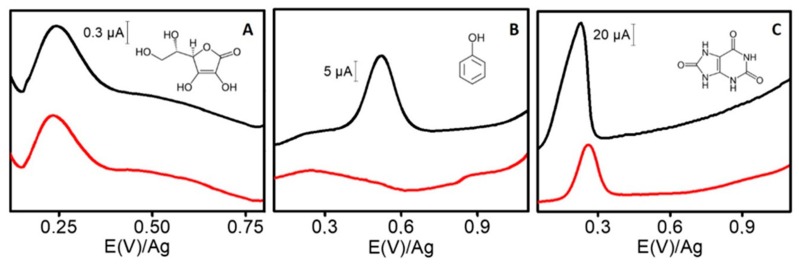
Voltammograms for 800 µM ascorbic acid (**A**), 800 µM phenol (**B**), and 500 µM uric acid (**C**). Red line: AdTDPV analysis, black line: DPV analysis. Conditions: as in Figure 4. Inset: Chemical structure of each compound.

As it is shown in Figure 6A, ascorbic acid yields a small peak in AdTDPV and DPV, but this peak occurs at +0.20 V, and it does not interfere with TET peak (electrochemical selectivity). Interestingly, in the case of phenol, it yields a peak in DPV but not in AdTDPV (see Figure 6B). This means that phenol does not strongly adsorb on ERGO-SPE, so this material acts as selective adsorbent of TET respect phenol (adsorptive selectivity). Indeed, in ERGO, not only π–π interaction occurs with the analytes, but hydrogen bonding is possible too [29,30]. Therefore, the higher number of delocalized π-electrons, and hydroxyl, carbonyl, and amine groups present in TET structure respect to phenol, provide a stronger interaction with ERGO surface. In summary, no significant changes were observed in TET quantitative recoveries (97% and 104%, 80 µM) in the presence of ascorbic acid and phenol, using a 1:10 TET/interference ratio.

However, uric acid (at 500 µM as common concentration in urine samples) [31] yields an intense peak in both techniques (AdTDPV and DPV) (see Figure 6C). The strong absorption ability of uric acid on the ERGO-SPE surface proved to have a clear impact on the AdTDPV of the TETs (recovery 43% for 80 µM TET). Like TET, uric acid presents a π–electron resonance in its double ring and also carbonyl and amine groups, so it causes a strong π–π stacking and hydrogen bonding interaction between the uric acid and ERGO. In consequence, an additional study was carried out to establish the maximum concentration of uric acid that does not interfere in the measurement. It was estimated that concentrations of uric acid above 160 µM may interfere in TET determination.

**Figure 7:**
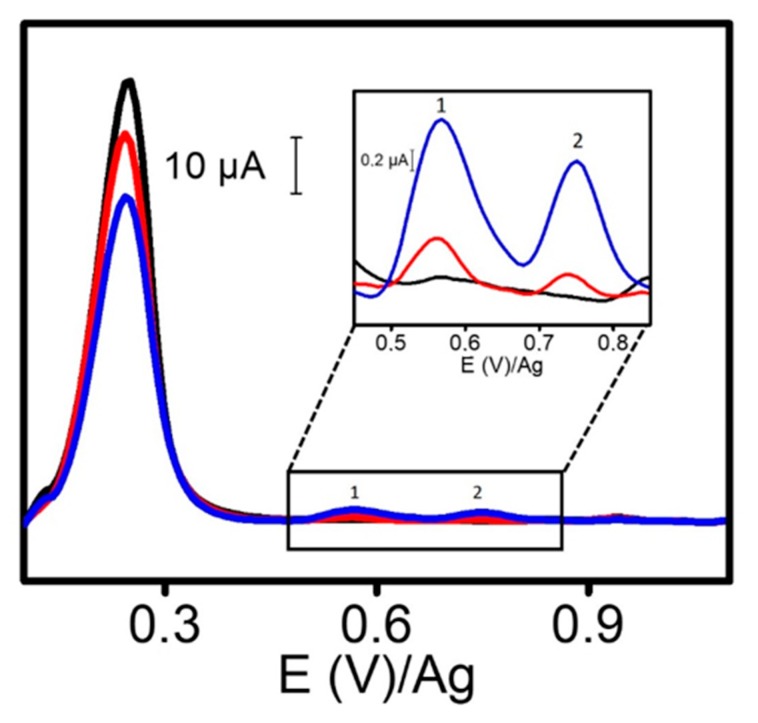
AdTDPV of skim milk (black), spiked with 30 µM TET (red) and 70 µM TET (blue). Conditions as in Figure 5. Peaks 1 and 2 belong to TET.

Then, the analysis of milk and river water samples was carried out. Milk samples were pretreated for eliminating proteins (described in Section 2.4). Figure 7 showed AdTDPV voltammograms for non-spiked and TET spiked skim milk. In spite of the presence of an unknown intense peak around +0.20 V, TET was easily determined. In fact, recovery yields were acceptable in both analyzed samples and in both assayed concentration levels as is listed in Table 2. These results demonstrated that this method is adequate for the assessment of total tetracyclines in food and environmental samples.

## 4. Conclusions

Governing both adsorptive and electrochemical selectivity, an electrochemical sensor based on AdTDPV-ERGO was developed for total tetracycline determination in milk and river samples using disposable screen-printed electrodes. The sensor capability to assess the total tetracyclines was demonstrated since individual tetracyclines and mixtures exhibited identical analytical sensitivity since no statistically differences were found when the calibration slopes were carefully compared (*p* < 0.05). The approach took only 6 min and yielded quantitative recoveries.

AdTDPV-ERGO-SPEs combine the interesting properties of AdTDPV (adsorption control), graphene (high adsorption capacity and excellent electrochemical transduction), and SPE technology (disposability, “in situ” analysis capabilities and low cost), making them a promising approach for electrochemical sensing of other phenol-based target molecules with a high significance.

## Figures and Tables

**Table 1:** DPV and AdTDPV analysis TET on SPE and ERGO-SPE transducers ^1^.

Electrode	Peak Potential (V)	DPV Peak Height (×10^8^ A)	AdTDPV Peak Height (×10^8^ A)
SPE	0.58	175	4.0
ERGO-10	0.54	23.9	131
ERGO-20	0.55	28.7	218

**^1^** 60 µM TET in phosphate buffer (50 mM, pH 6.0).

**Table 2:** Spiked sample analysis ^1^.

Sample	TET Added (µM)	Found (µM)	Recovery (%)
Milk	30	28	95 ± 5
	70	71	102 ± 21
River water	30	33	110 ± 10
	70	66	94 ± 9

^1^ Results are given as mean values ± SD, *n* = 3).

## Supplementary Materials

The following are available online at https://www.mdpi.com/1424-8220/20/1/76/s1, Figure S1: Comparative between cyclic voltammetry and AdTDPV analysis for tetracycline using ERGO-20, Figure S2: AdTDPV of TET 0.5 mM on 20 cycles ERGO-SPE using pulse amplitudes from 25 mV to 150 mV, Figure S3: Signal intensity of 0.5 mM TET by AdTDPV at different pHs, Figure S4: AdTDPV voltammograms of TET at different concentrations.

## Abbreviations

AdTDPV	adsorptive transfer stripping differential pulse voltammetry
AdSDPV	adsorptive stripping differential pulse voltammetry
CTC	chlortetracycline hydrochloride
DOX	doxycycline hydrochloride
DPV	differential pulse voltammetry
ERGO	electrochemically reduced graphene oxide
GO	graphene oxide
OTC	oxytetracycline hydrochloride
SEM	scanning electron microscopy
SPE	carbon screen-printed electrode
TET	tetracycline hydrochloride

## References

[1] Garcia C.D., Crevillen A.G., Escarpa A. (2018). Carbon-Based Nanomaterials in Analytical Chemistry.

[2] Martín A., Escarpa A. (2014). Graphene: The cutting-edge interaction between chemistry and electrochemistry. TrAC Trends Anal. Chem..

[3] Pérez-López B., Merkoçi A. (2012). Carbon nanotubes and graphene in analytical sciences. Microchim. Acta.

[4] Ibrahim W.A.W., Nodeh H.R., Sanagi M.M. (2016). Graphene-Based Materials as Solid Phase Extraction Sorbent for Trace Metal Ions, Organic Compounds, and Biological Sample Preparation. Crit. Rev. Anal. Chem..

[5] Sitko R., Zawisza B., Malicka E. (2013). Graphene as a new sorbent in analytical chemistry. TrAC Trends Anal. Chem..

[6] Khodaee N., Mehdinia A., Esfandiarnejad R., Jabbari A. (2016). Ultra trace analysis of PAHs by designing simple injection of large amounts of analytes through the sample reconcentration on SPME fiber after magnetic solid phase extraction. Talanta.

[7] Wu X.J., Wang G.N., Yang K., Liu H.Z., Wang J.P. (2017). Determination of Tetracyclines in Milk by Graphene-Based Solid-Phase Extraction and High-Performance Liquid Chromatography. Anal. Lett..

[8] Yan H., Sun N., Liu S., Row K.H., Song Y. (2014). Miniaturized graphene-based pipette tip extraction coupled with liquid chromatography for the determination of sulfonamide residues in bovine milk. Food Chem..

[9] Justino C.I.L., Gomes A.R., Freitas A.C., Duarte A.C., Rocha-Santos T.A.P. (2017). Graphene based sensors and biosensors. TrAC Trends Anal. Chem..

[10] Cinti S., Arduini F. (2017). Graphene-based screen-printed electrochemical (bio)sensors and their applications: Efforts and criticisms. Biosens. Bioelectron..

[11] Taleat Z., Khoshroo A., Mazloum-Ardakani M. (2014). Screen-printed electrodes for biosensing: A review (2008–2013). Microchim. Acta.

[12] Kushikawa R.T., Silva M.R., Angelo A.C.D., Teixeira M.F.S. (2016). Construction of an electrochemical sensing platform based on platinum nanoparticles supported on carbon for tetracycline determination. Sens. Actuators B Chem..

[13] Chopra I., Roberts M. (2001). Tetracycline Antibiotics: Mode of Action, Applications, Molecular Biology, and Epidemiology of Bacterial Resistance. Microbiol. Mol. Biol. Rev..

[14] Pérez-Rodríguez M., Pellerano R.G., Pezza L., Pezza H.R. (2018). An overview of the main foodstuff sample preparation technologies for tetracycline residue determination. Talanta.

[15] Kumar M., Jaiswal S., Sodhi K.K., Shree P., Singh D.K. (2019). Antibiotics bioremediation: Perspectives on its ecotoxicity and resistance. Environ. Int..

[16] Lan L., Yao Y., Ping J., Ying Y. (2017). Recent advances in nanomaterial-based biosensors for antibiotics detection. Biosens. Bioelectron..

[17] Liu X., Huang D., Lai C., Zeng G., Qin L., Zhang C., Yi H., Li B., Deng R., Liu S. (2018). Recent advances in sensors for tetracycline antibiotics and their applications. TrAC Trends Anal. Chem..

[18] Gaudin V. (2017). Advances in biosensor development for the screening of antibiotic residues in food products of animal origin—A comprehensive review. Biosens. Bioelectron..

[19] Kesavan S., Kumar D.R., Lee Y.R., Shim J.J. (2017). Determination of tetracycline in the presence of major interference in human urine samples using polymelamine/electrochemically reduced graphene oxide modified electrode. Sens. Actuators B Chem..

[20] Sun X., Ji Z., Xiong M., Chen W. (2017). The Electrochemical Sensor for the Determination of Tetracycline Based on Graphene /L-Cysteine Composite Film. J. Electrochem. Soc..

[21] Wong A., Scontri M., Materon E.M., Lanza M.R.V., Sotomayor M.D.P.T. (2015). Development and application of an electrochemical sensor modified with multi-walled carbon nanotubes and graphene oxide for the sensitive and selective detection of tetracycline. J. Electroanal. Chem..

[22] Paleček E., Bartošík M. (2012). Electrochemistry of Nucleic Acids. Chem. Rev..

[23] Jampasa S., Siangproh W., Duangmal K., Chailapakul O. (2016). Electrochemically reduced graphene oxide-modified screen-printed carbon electrodes for a simple and highly sensitive electrochemical detection of synthetic colorants in beverages. Talanta.

[24] Hui K.H., Ambrosi A., Pumera M., Bonanni A. (2016). Improving the Analytical Performance of Graphene Oxide towards the Assessment of Polyphenols. Chem. A Eur. J..

[25] Luo Y., Xu J., Li Y., Gao H., Guo J., Shen F., Sun C. (2015). A novel colorimetric aptasensor using cysteamine-stabilized gold nanoparticles as probe for rapid and specific detection of tetracycline in raw milk. Food Control.

[26] Moraes F.C., Freitas R.G., Pereira R., Gorup L.F., Cuesta A., Pereira E.C. (2015). Coupled electronic and morphologic changes in graphene oxide upon electrochemical reduction. Carbon.

[27] Šalplachta J., Horká M., Růžička F., Šlais K., Vykydalová M., Kubesová A., Holá V., Dvořáčková M. (2014). CIEF separation, UV detection, and quantification of ampholytic antibiotics and bacteria from different matrices. Anal. Bioanal. Chem..

[28] Sierra T., González M.C., Moreno B., Crevillen A.G., Escarpa A. (2018). Total α1-acid glycoprotein determination in serum samples using disposable screen-printed electrodes and osmium (VI) as electrochemical tag. Talanta.

[29] Hou X., Wang X., Sun Y., Wang Y., Guo Y. (2017). Graphene oxide for solid-phase extraction of bioactive, phenolic acids. Anal. Bioanal. Chem..

[30] Martín A., Hernández-Ferrer J., Vázquez L., Martínez M.T., Escarpa A. (2014). Controlled chemistry of tailored graphene nanoribbons for electrochemistry: A rational approach to optimizing molecule detection. RSC Adv..

[31] Abellán-Llobregat A., Vidal L., Rodríguez-Amaro R., Berenguer-Murcia A., Canals A., Morallón E. (2017). Au-IDA microelectrodes modified with Au-doped graphene oxide for the simultaneous determination of uric acid and ascorbic acid in urine samples. Electrochim. Acta.

