# Supplementary material for: Electrochemically Reduced Graphene Oxide-Based Screen-Printed Electrodes for Total Tetracycline Determination by Adsorptive Transfer Stripping Differential Pulse Voltammetry
Source: Sensors (Basel). 2019 Dec 21;20(1):76. doi: 10.3390/s20010076 (PMC6983226; doi:10.3390/s20010076)
Supplement: Supplementary file 1 [file sensors-20-00076-s001.pdf]

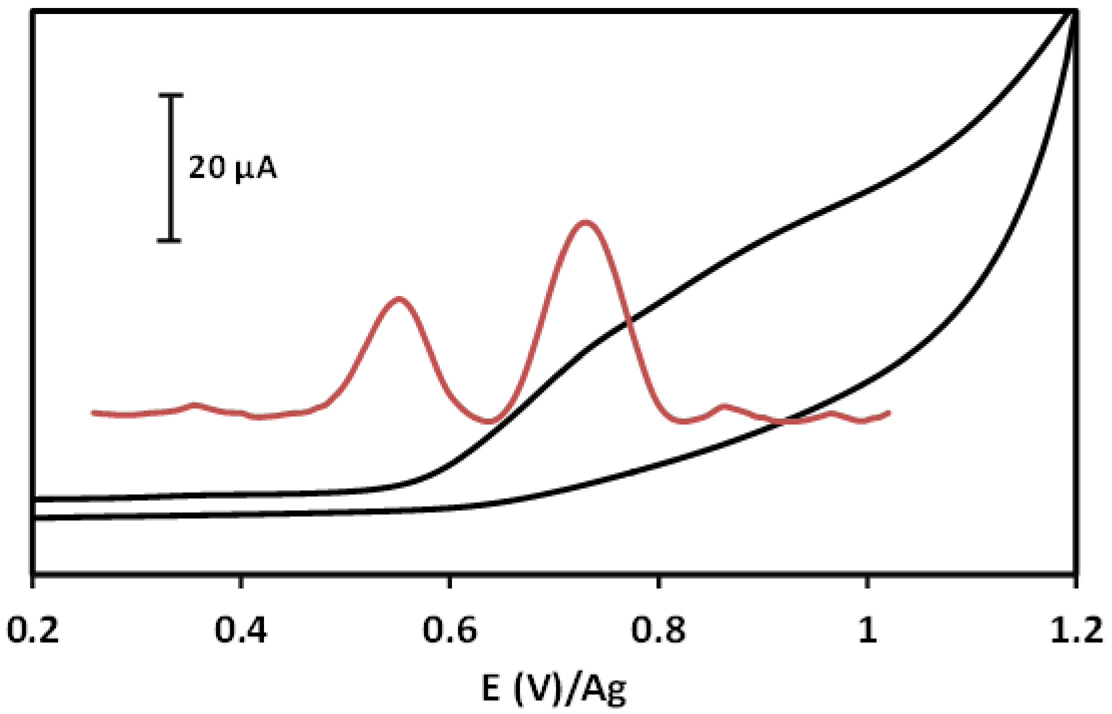

Fig S1 Comparative between cyclic voltammetry (1.25 mM TET, black line) and AdTDPV (80 µM TET, red line, this signal was multiplied by 6 for illustrative purpose) analysis for tetracycline using ERGO-20. Conditions for cyclic voltammetry: phosphate buffer 50 mM pH 6, scan rate 100 mV s^{-1}. Conditions for AdTDPV: phosphate buffer 50 mM pH 6, pulse amplitude 125 mV, pulse time 0.05 s, interval time 0.3 s, step potential 8 mV, adsorption time 5 min.

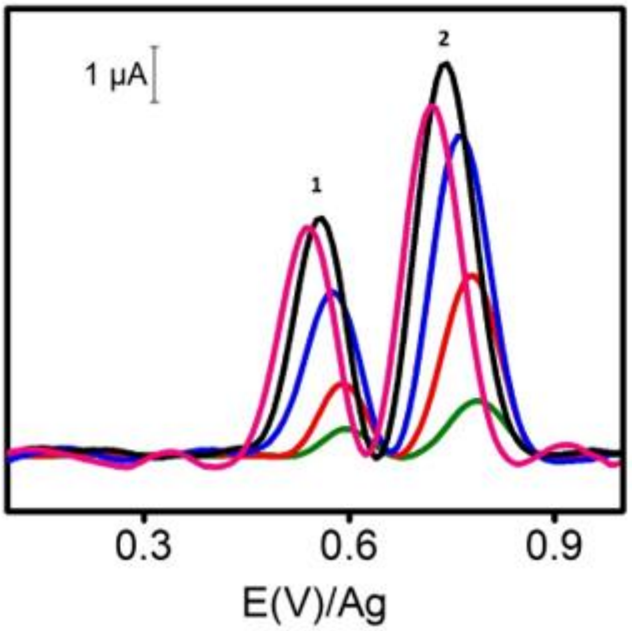

Fig. S2 AdTDPV of TET 0.5 mM on 20 cycles ERGO-SPE using pulse amplitudes of 25 mV (green), 50 mV (red), 100 mV (blue), 125 mV (black) and 150 mV (pink). Conditions:

Phosphate buffer pH 6 50 mM, pulse time 0.05 s, interval time 0.3 s, step potential 8 mV, adsorption time 5 min

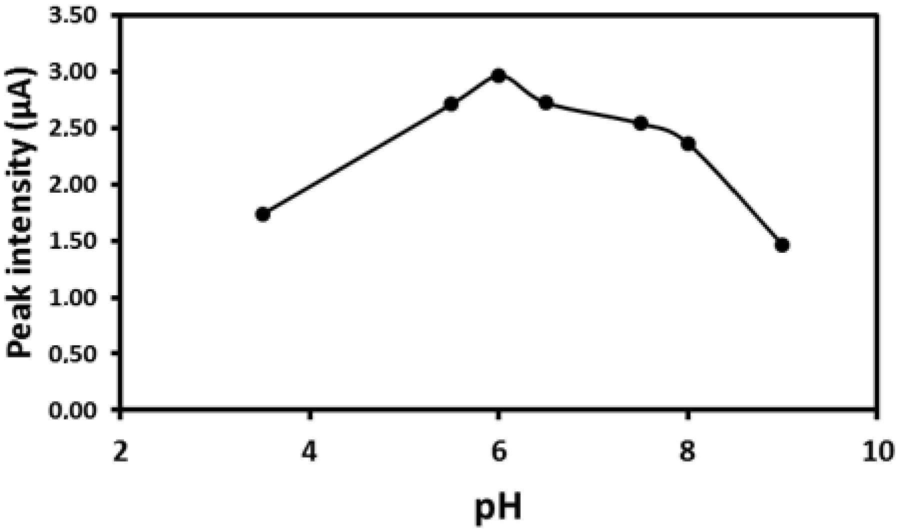

Fig. S3 Peak 1 intensity of 0.5 mM TET by AdTDPV at different pHs. Conditions: phosphate buffer 50 mM, pulse amplitude 125 mV, pulse time 0.05 s, interval time 0.3 s, step potential 8 mV, adsorption time 5 min

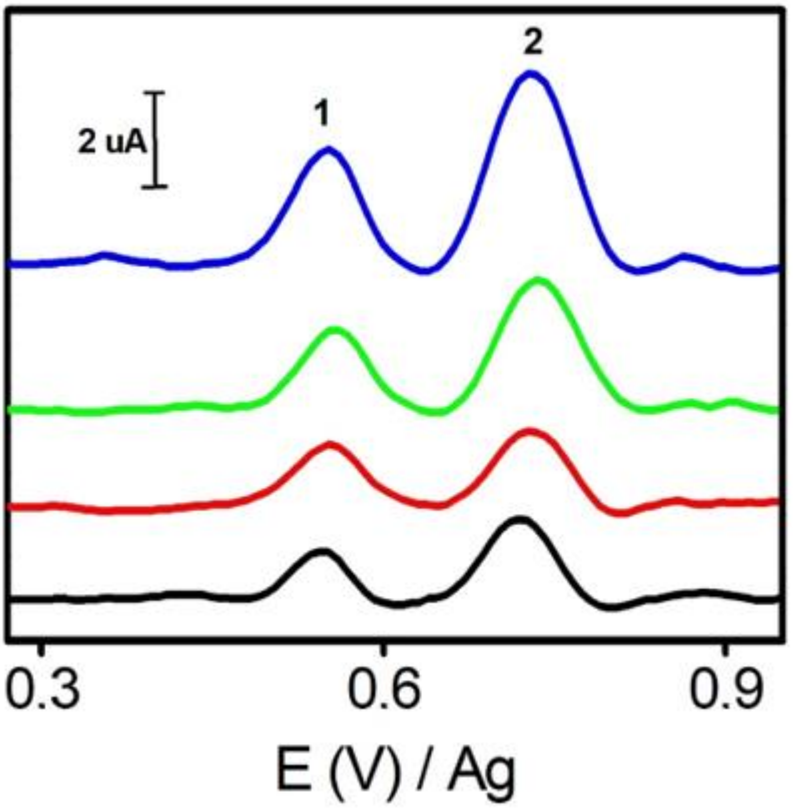

Fig. S4 AdTDPV voltammograms of TET at different concentrations (black line 20 µM, red line 40 µM, green line 60 µM and blue line 80 µM. Conditions:

phosphate buffer 50 mM pH 6, pulse amplitude 125 mV, pulse time 0.05 s, interval time 0.3 s, step potential 8 mV, adsorption time 5 min
